# Supplementary material for: A temporal single cell transcriptome atlas of zebrafish anterior segment development
Source: Sci Rep. 2023 Apr 6;13:5656. doi: 10.1038/s41598-023-32212-4 (PMC10079958; doi:10.1038/s41598-023-32212-4)

SUPPLEMENTAL FIGURES

Figure S1: Two color FWISH examination of AS marker gene co-expression.
A) Two color FWISH depicting co-expression of krt4(red)/krt5(green), krt91(green)/krt4(red) and icn2(green)/krt4(red) at 120hpf. DAPI (blue) was used to stain the nuclei. Volume projections in lateral view and single section dorsal views from 3D confocal stacks are displayed. White arrows indicate example regions of co-expression. **B)** Two color FWISH depicting co-expression of pmp22a(red)/ctnnb2(green) at 120hpf. DAPI (blue) was used to stain the nuclei. Volume projections in lateral view and single section dorsal views from 3D confocal stacks are displayed. **C)** Two color FWISH depicting co-expression of lum(red)/dcn(green) at 120hpf. DAPI (blue) was used to stain the nuclei. Volume projections in lateral view and single section dorsal views from 3D confocal stacks are displayed. **D)** Two color FWISH depicting co-expression of hmgb2b(green)/scinla(red), hmnb2(green)/scinla(red) and myoc(green)/scinla(red) at 120hpf. DAPI (blue) was used to stain the nuclei. Volume projections in lateral view and single section dorsal views from 3D confocal stacks are displayed. Scale bar = 100μm.

Figure S2: Marker gene expression distribution in AS associated clusters at 48hpf.
Heatmap distribution of canonical AL1 (hmgn2, hmgb2b, mcm7, pcna), AL2 (scinla, lxn), CEp1 (krt5, krt91, epcam), CEp2 (krt4, icn2, cyt1), CEn (pmp22a, ctnnb2) or CSt (dcn, lum) markers for AS associated clusters at 48hpf.

Figure S3: Marker gene expression distribution in AS associated clusters at 72hpf.
Heatmap distribution of canonical AL1 (hmgn2, hmgb2b, mcm7, pcn1), AL2 (scinla, lxn), CEp1 (krt5, krt91, epcam), CEp2 (krt4, icn2, cyt1), CEn (pmp22a, ctnnb2) or CSt (dcn, lum) markers for AS associated clusters at 72hpf.

Figure S4: Marker gene expression distribution in AS associated clusters at 96hpf.
Heatmap distribution of canonical AL1 (hmgn2, hmgb2b, mcm7, pcna), AL2 (scinla, lxn), CEp1 (krt5, krt91, epcam), CEp2 (krt4, icn2, cyt1), CEn (pmp22a, ctnnb2) or CSt (dcn, lum) markers for AS associated clusters at 96hpf.

Figure S5: Marker gene expression distribution in AS associated clusters at 120hpf. Heatmap distribution of canonical AL1 (hmgn2, hmgb2b, mcm7, pcn1), AL2 (scinla, lxn), CEp1 (krt5, krt91, epcam), CEp2 (krt4, icn2, cyt1), CEn (pmp22a, ctnnb2) or CSt (dcn, lum) markers for AS associated clusters at 120hpf.

Figure S6: Marker gene expression distribution in AS associated clusters at 144hpf. Heatmap distribution of canonical AL1 (hmgn2, hmgb2b, mcm7, pcna), AL2 (scinla, lxn), CEp1 (krt5, krt91, epcam), CEp2 (krt4, icn2, cyt1), CEn (pmp22a, ctnnb2) or CSt (dcn, lum) markers for AS associated clusters at 144hpf.

Figure S7: Corneal and annular ligament marker gene expression over time. **A)** Average mean expression measurements from CEp1 clusters for genes *krt5 and krt91* over developmental time. **B)** Average mean expression measurements from CEp2 clusters for *krt4* and *icn2* over developmental time. **C)** Average mean expression measurements from AL1 clusters for genes *myoc, hmgn2* and *hmgb2b* over developmental time. **D)** Average mean expression measurements from AL2+CEn clusters for genes *scinla, ctnnb2, pmp22a* and *lxn* over developmental time. **E)** Average mean expression measurements from CEn clusters for genes *ctnnb2* and *pmp22a* over developmental time. **F)** Average mean expression measurements from CEn+CSt clusters for genes *dcn, lum, ctnnb2* and *pmp22a* over developmental time.

Figure S8: Verification of Pigment cell expression patterns. WISH expression analysis of select pigment cell associated genes at 24, 72 and 120hpf displayed in dorsal and lateral views.

Figure S9: Gene expression distribution in the pseudotime analysis of the combined datasets. Heatmap distribution of (A) sparc, lgals1l1, (B) col1a2, col4a5, col1a1b, (C) anxa1c, pnp5a, (D) fabp11a, (E) hgd, si:ch211-251b21.1 and (F) fmoda projected onto the dataset, including the aggregated data of 48hpf, 72hpf, 96hpf, 120hpf and 144hpf.

Figure S10: Two color FWISH examination of corneal epithelium marker expression
Two color FWISH depicting co-expression of krt4(red)/zgc:92380(green), sparc(green)/krt4(red), pfn1(green)/krt4(red), epcam(green)/krt4(red) and col1a1b(green)/krt4(red) at 120hpf. DAPI (blue) was used to stain the nuclei. Volume projections in lateral view and single section dorsal views from 3D confocal stacks are displayed. Scale bar = 100μm.

Figure S11: Two color FWISH examination of annular ligament marker expression
A) Two color FWISH depicting co-expression of nusap1(green)/scinla(red), frzb(green)/scinla(red), cxcr4b(green)/scinla, rrn2(green)/scinla(red) and cdh5(green)/scinla(red) at 120hpf. DAPI (blue) was used to stain the nuclei. Volume projections in lateral view and single section dorsal views from 3D confocal stacks are displayed. **B)** Two color FWISH depicting co-expression of mcm7(green)/scinla(red), vim(green)/scinla(red), lxn(green)/scinla, fmoda(green)/scinla(red) and fabp11a(green)/scinla(red) at 120hpf. DAPI (blue) was used to stain the nuclei. Volume projections in lateral view and single section dorsal views from 3D confocal stacks are displayed. **C)** Two color FWISH depicting co-expression of stmn1a(green)/scinla(red), si:ch211-252b21.1(green)/scinla(red), hgd(green)/scinla, phgdh(green)/scinla(red) and cndp1(green)/scinla(red) at 120hpf. DAPI (blue) was used to stain the nuclei. Volume projections in lateral view and single section dorsal views from 3D confocal stacks are displayed. Scale bar = 100μm.

Figure S12: Conserved and novel CEp associated gene expression patterns. **A)** WISH expression analysis of mammalian corneal epithelium conserved CEp associated genes at 24, 72 and 120hpf displayed in dorsal and lateral views. **B)** WISH expression analysis of potentially novel CEp associated genes at 24, 72 and 120hpf displayed in dorsal and lateral views.

Figure S13: Conserved and novel AL associated gene expression patterns. **A)** WISH expression analysis of mammalian TM conserved AL associated genes at 24, 72 and

120hpf displayed in dorsal and lateral views. **B)** WISH expression analysis of potentially novel AL associated genes at 24, 72 and 120hpf displayed in dorsal and lateral views.

Supplemental Figure 1

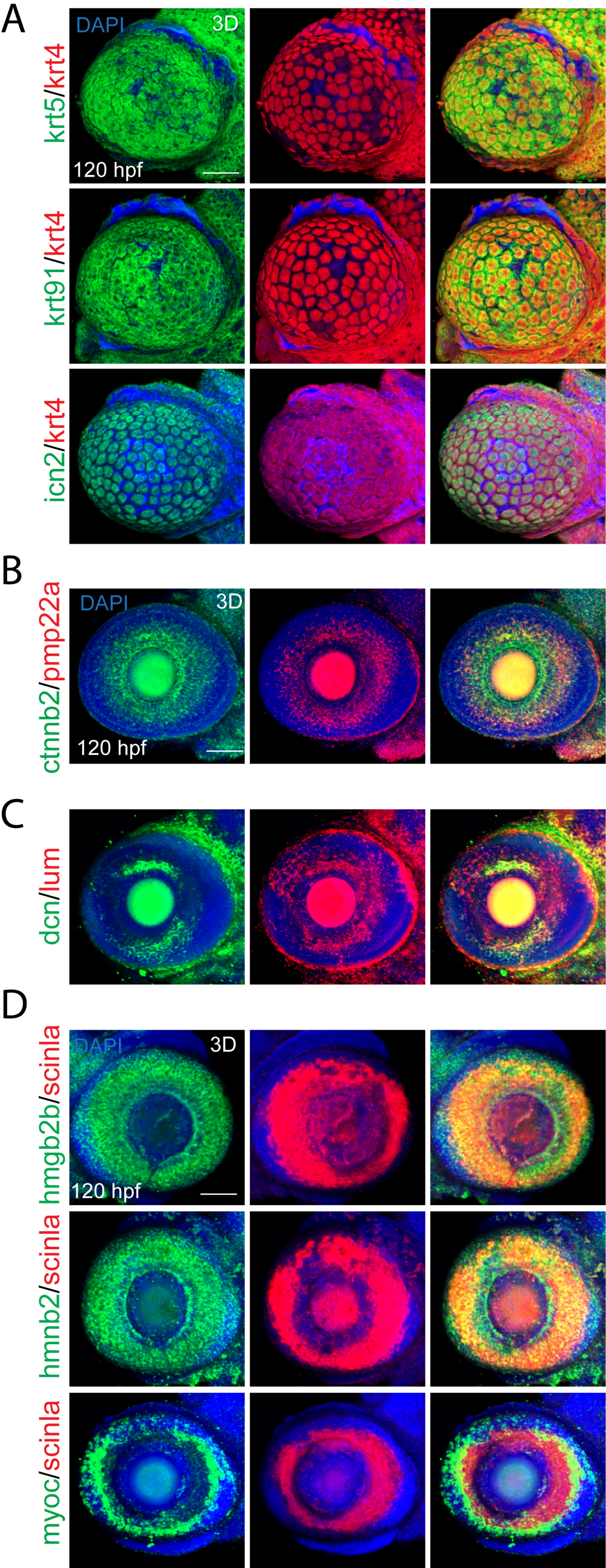

Supplemental Figure 2

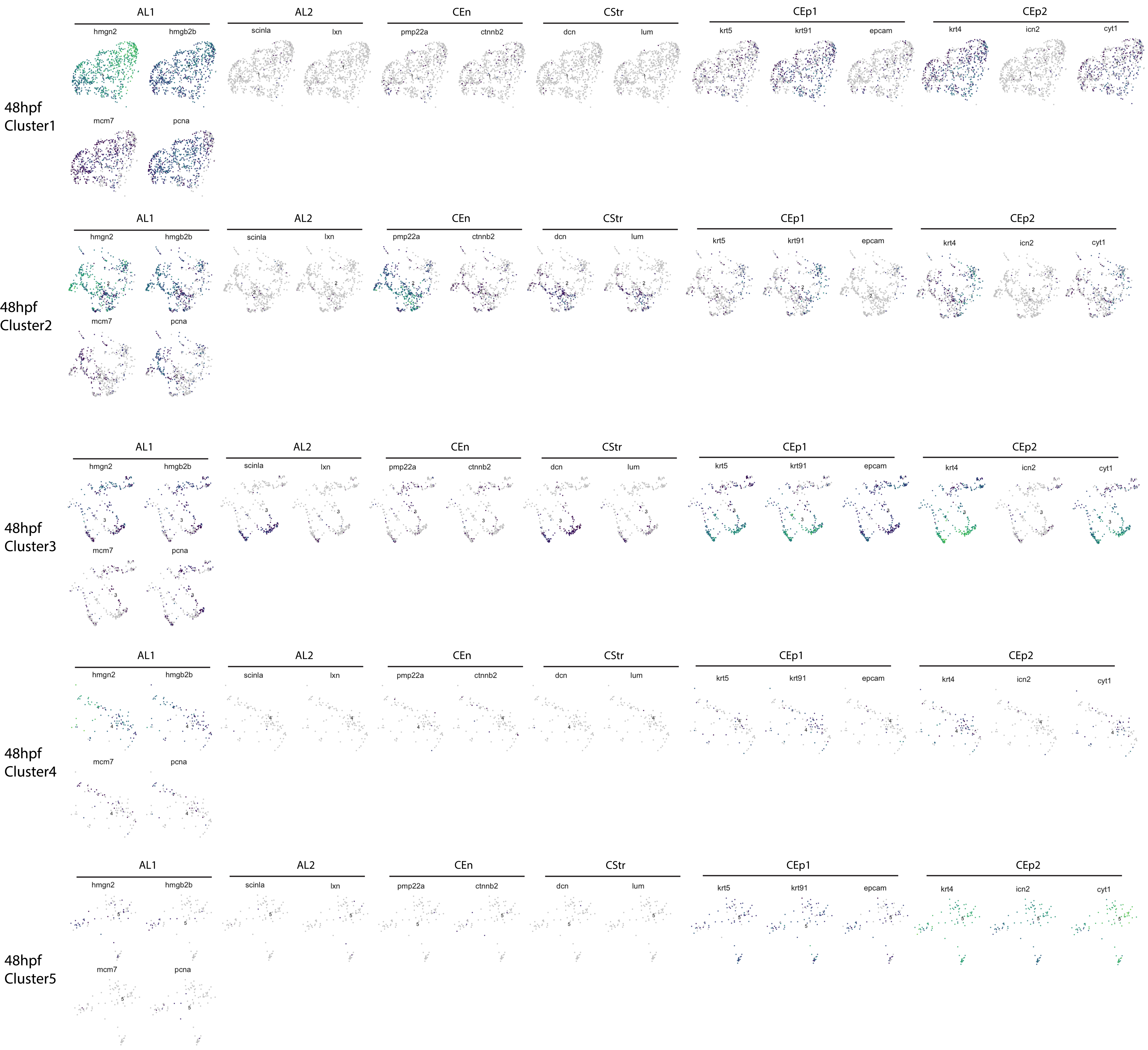

Supplemental Figure 3

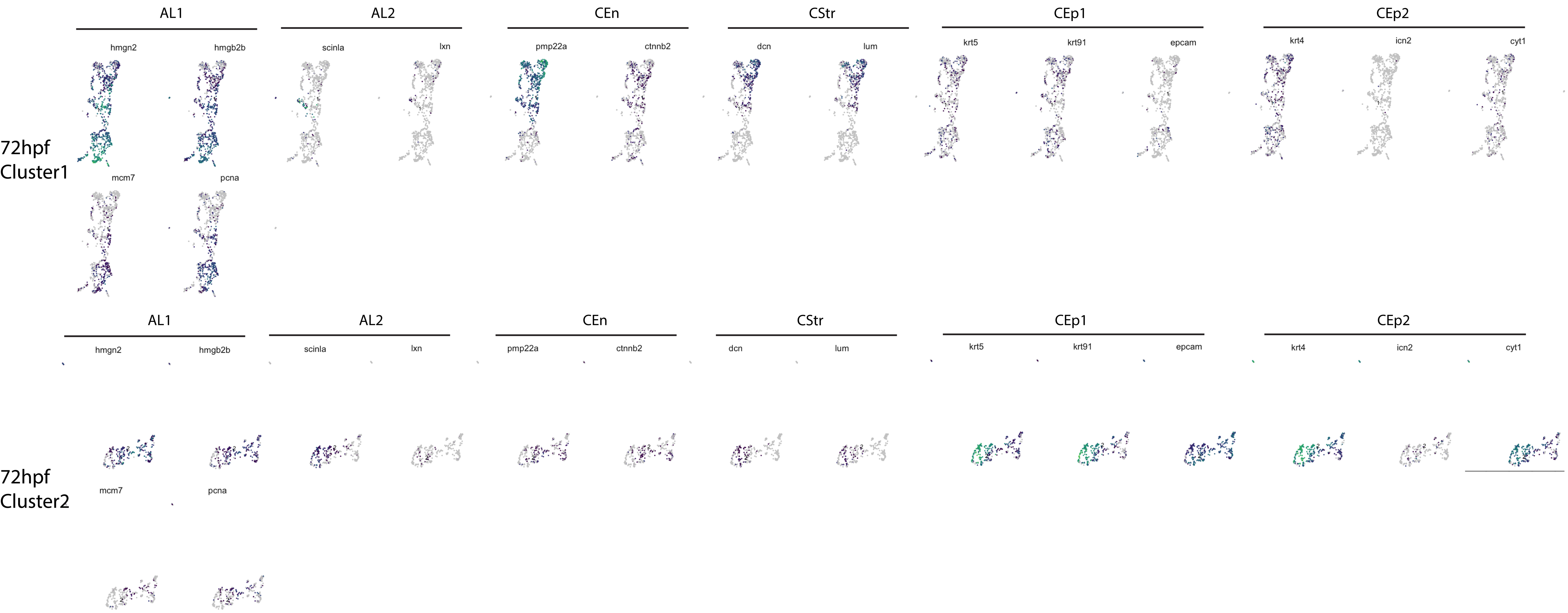

Supplemental Figure 4

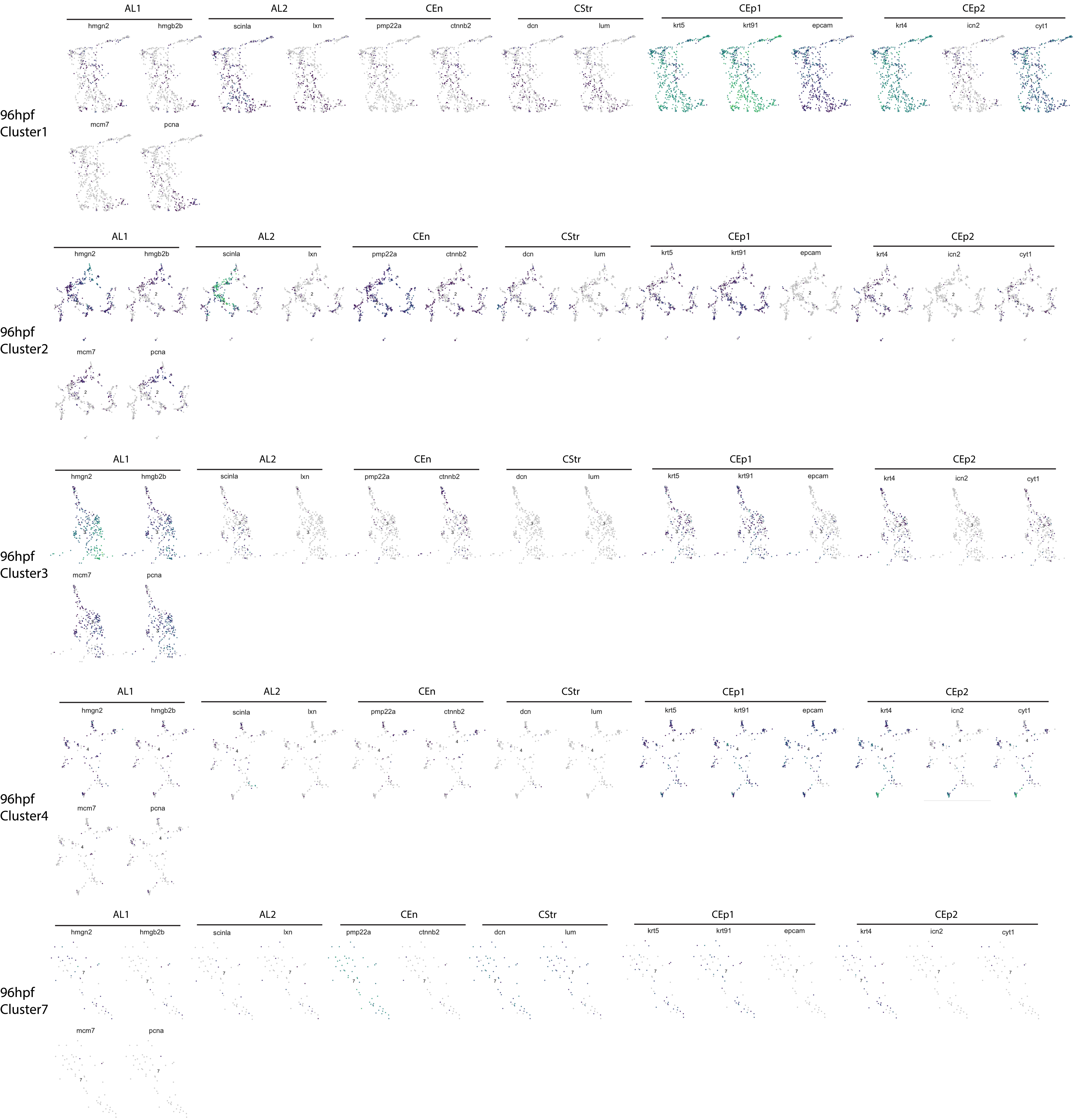

Supplemental Figure 5

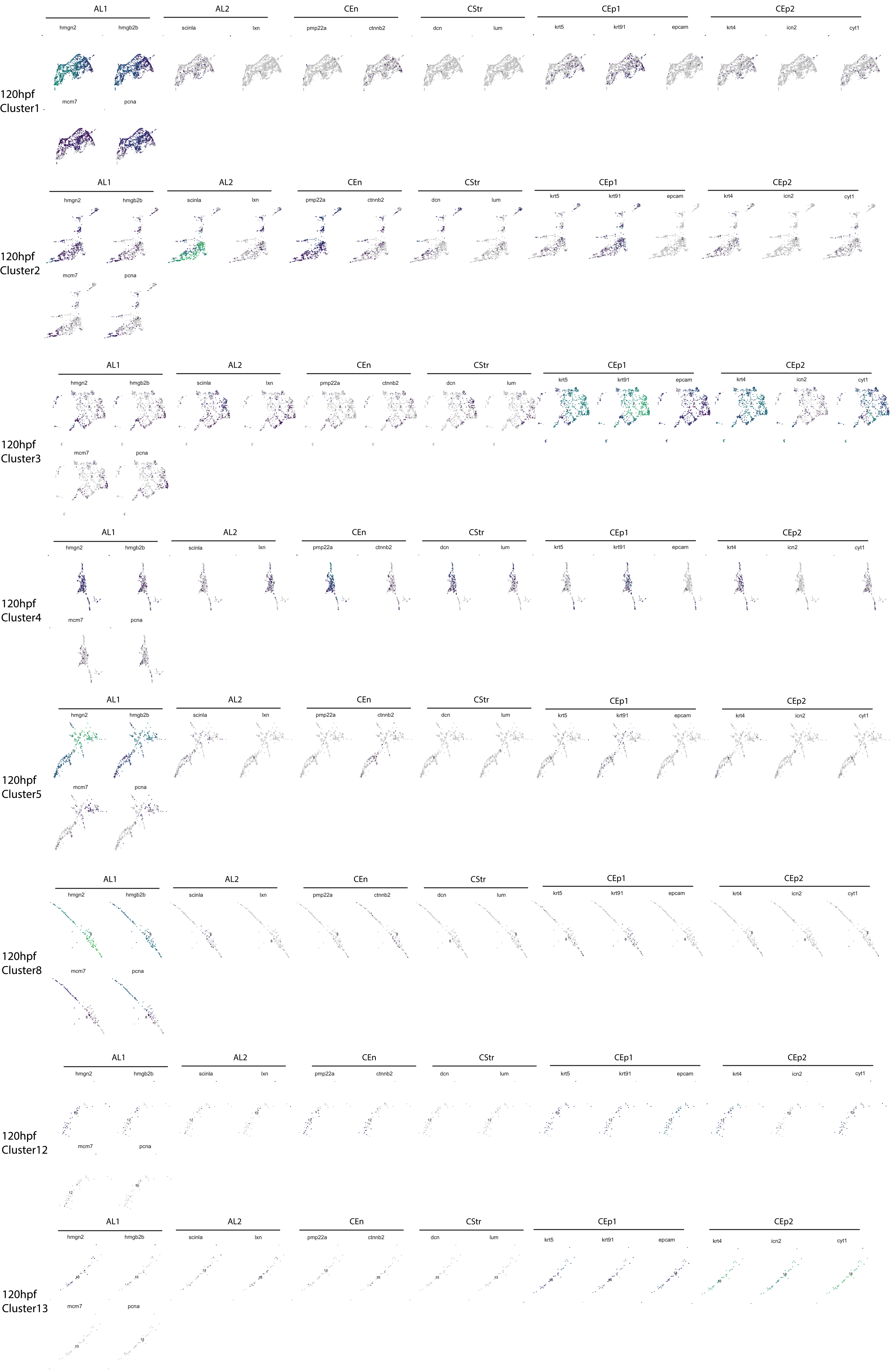

Supplemental Figure 6

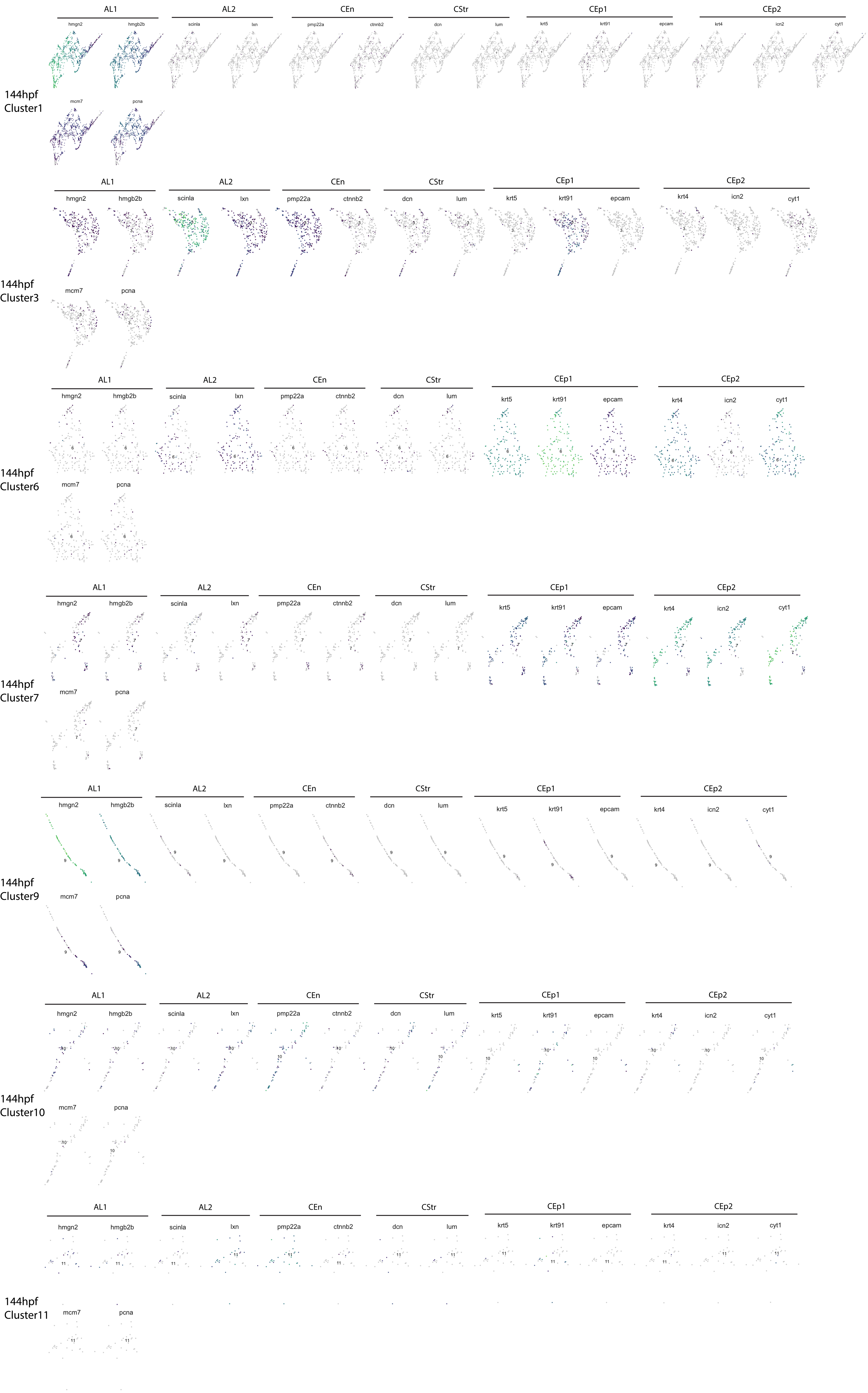

Supplemental Figure 7

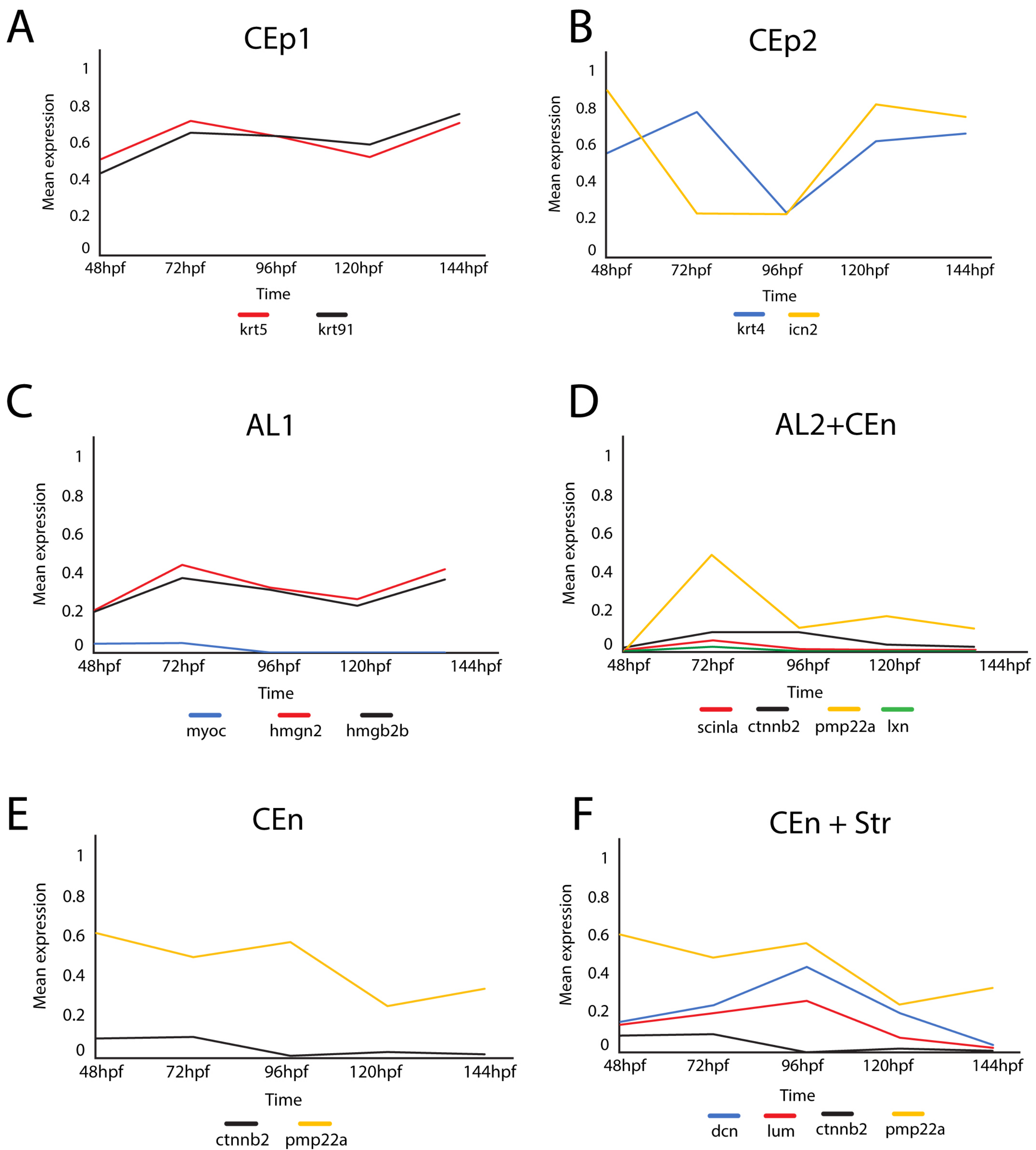

Supplementary Figure 8

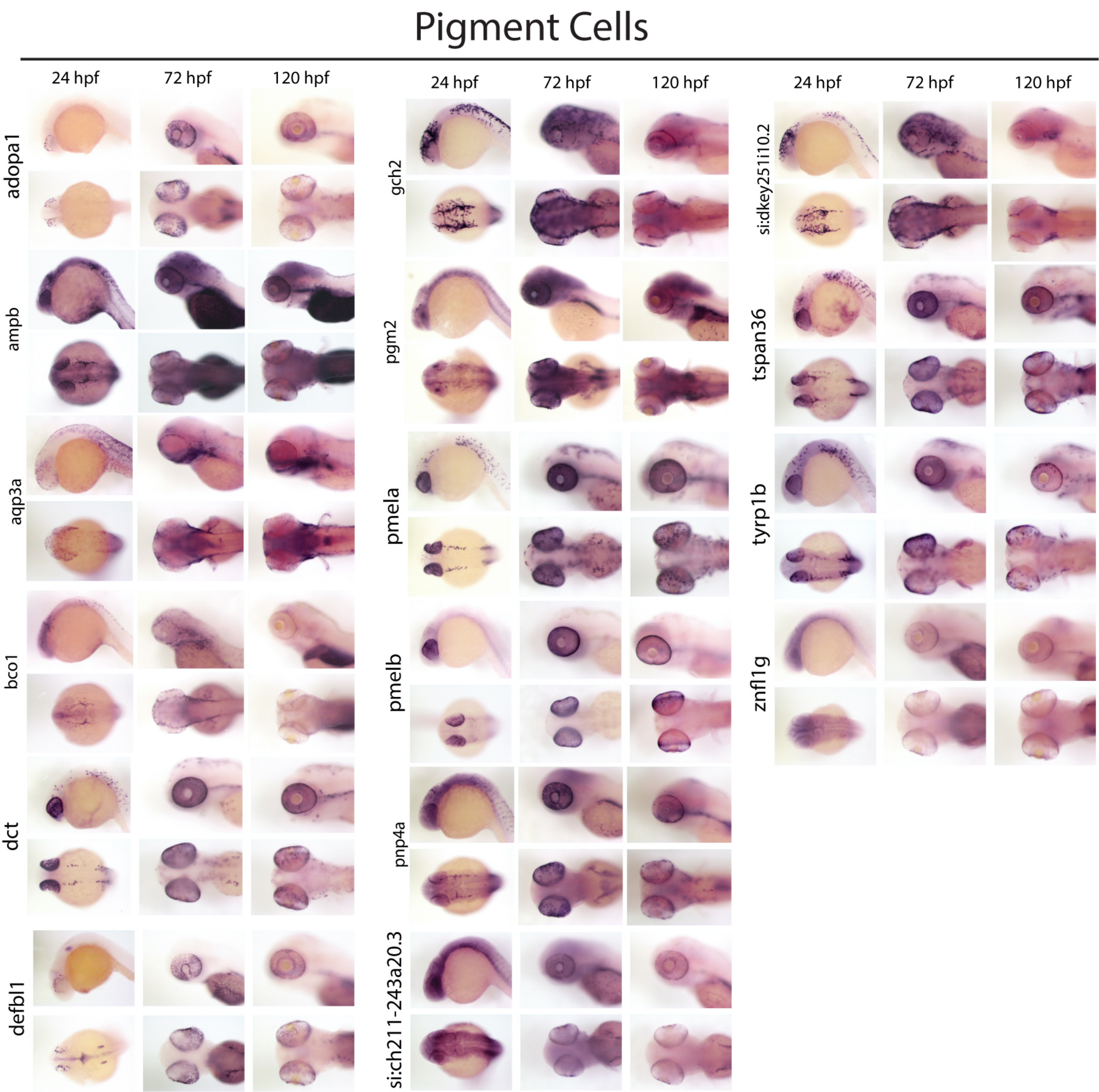

Supplemental Figure 9

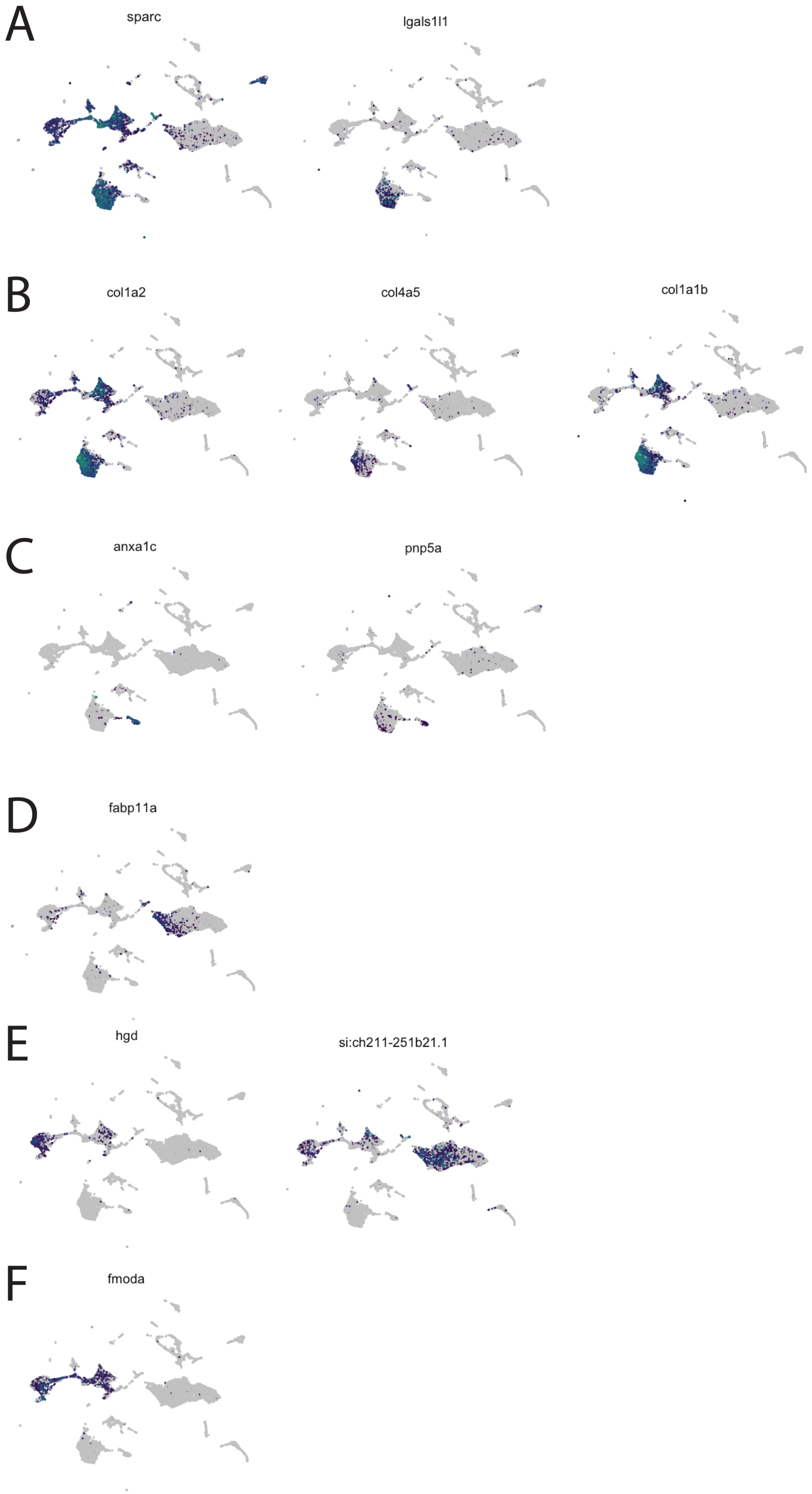

Supplementary Figure 10

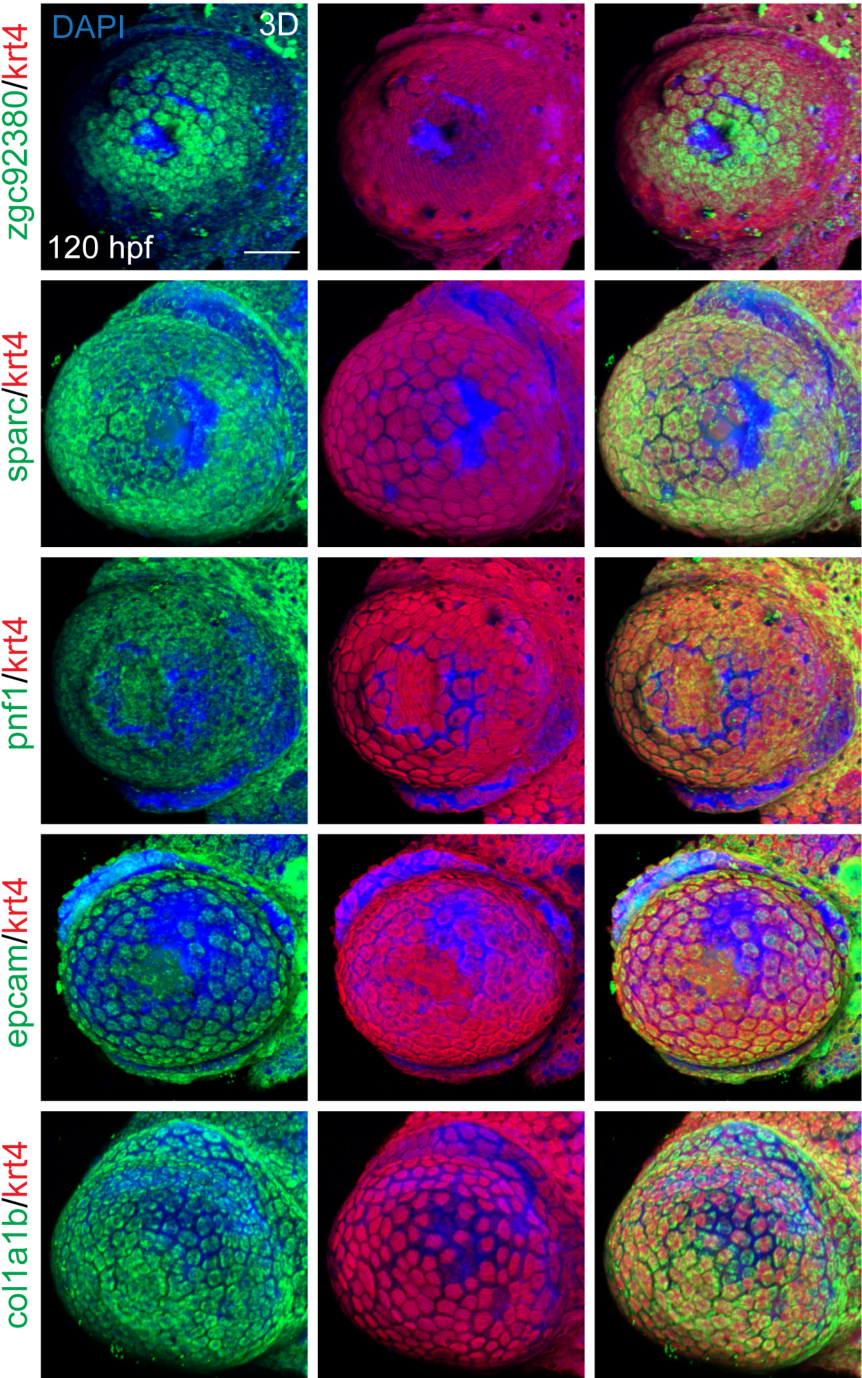

Supplementary Figure 11

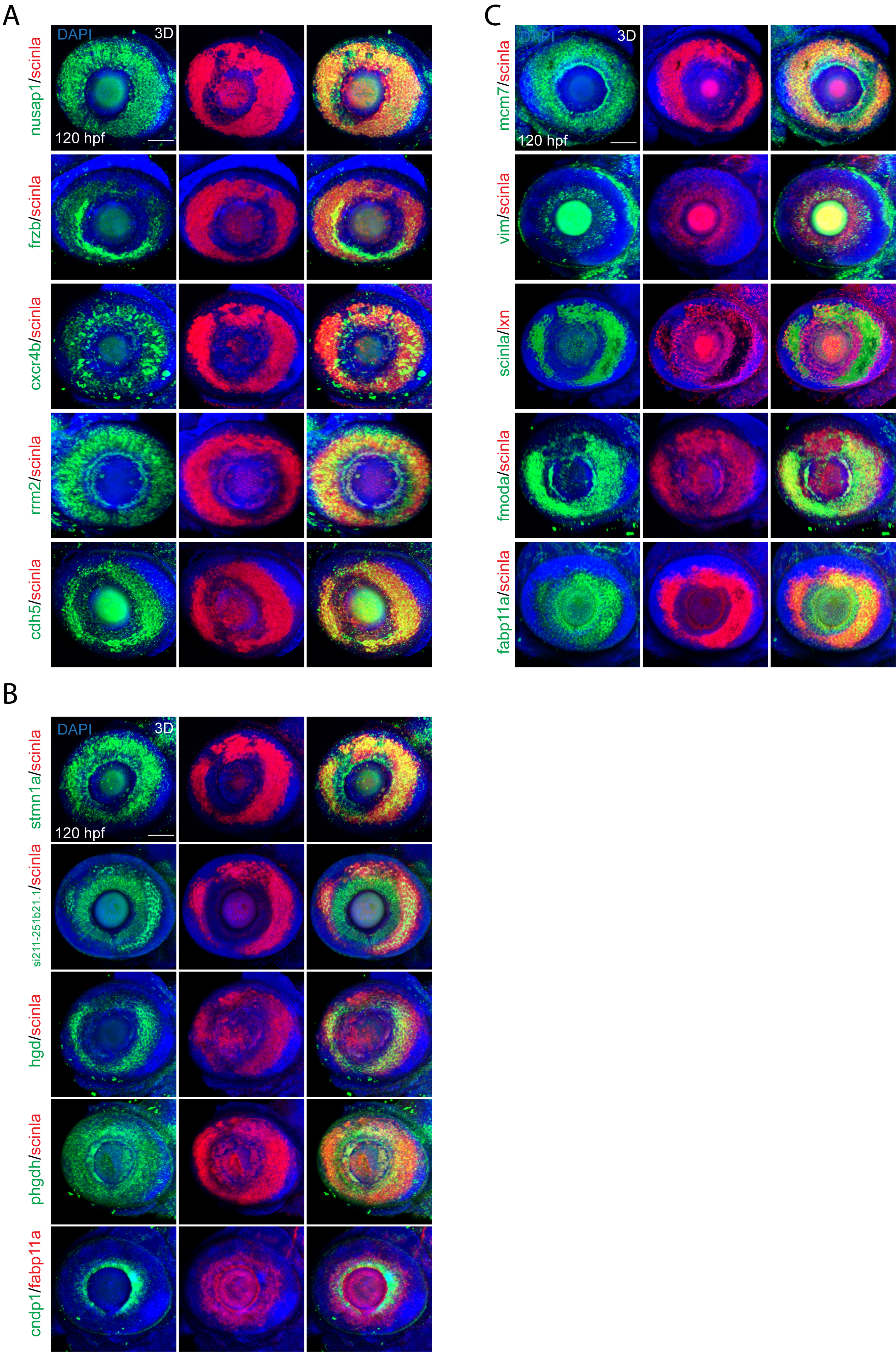

Supplemental Figure 12

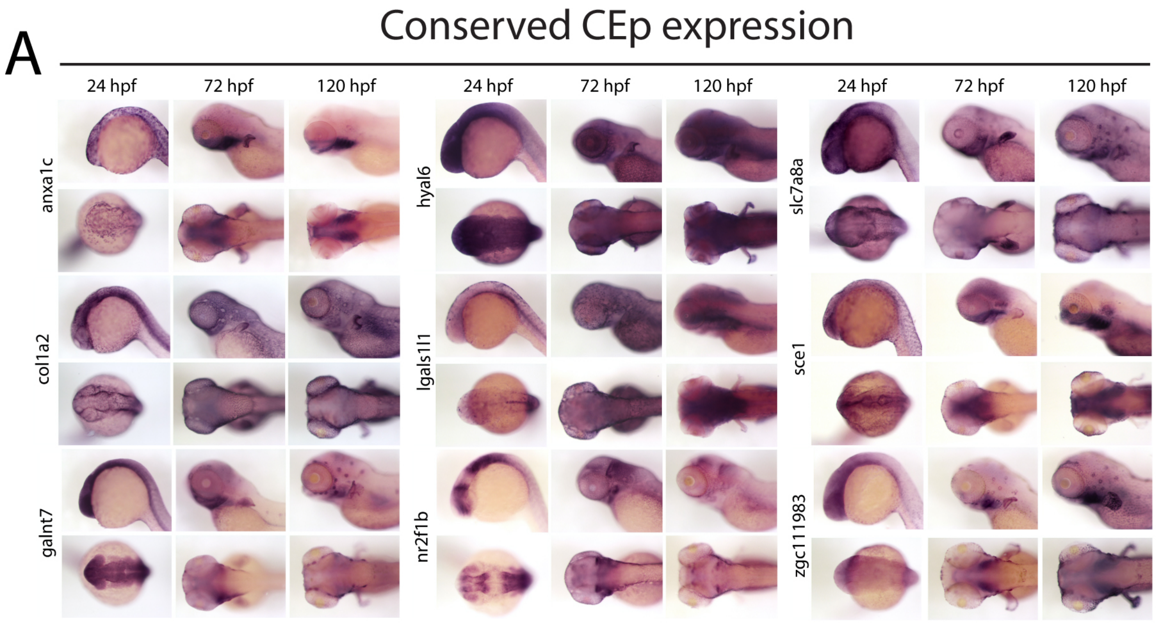

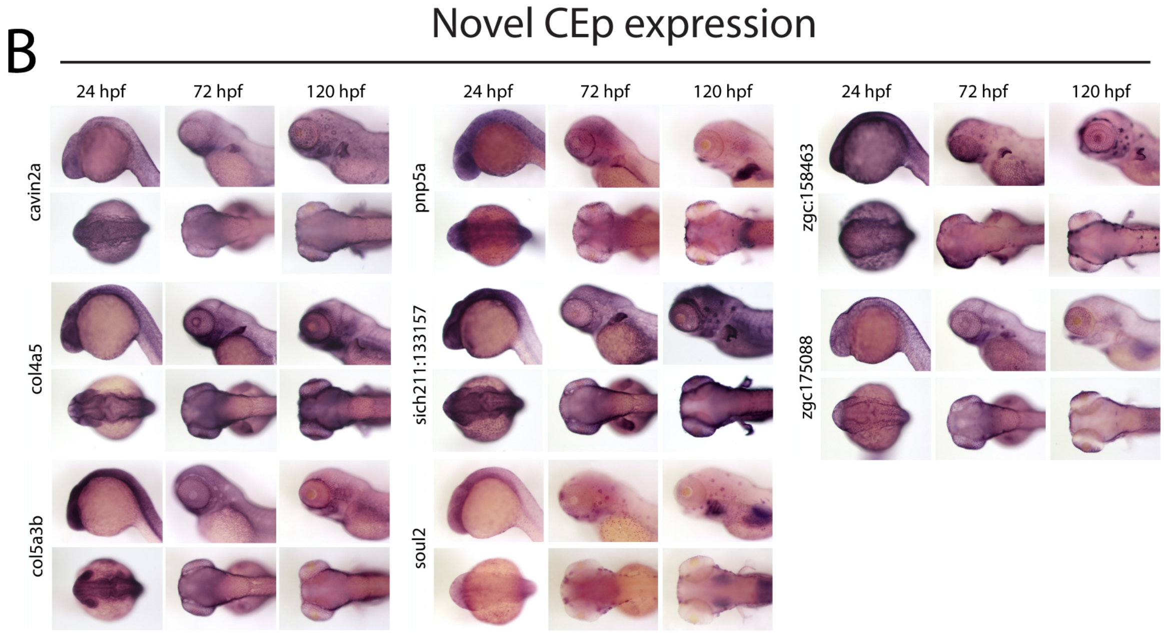

Supplemental Figure 13

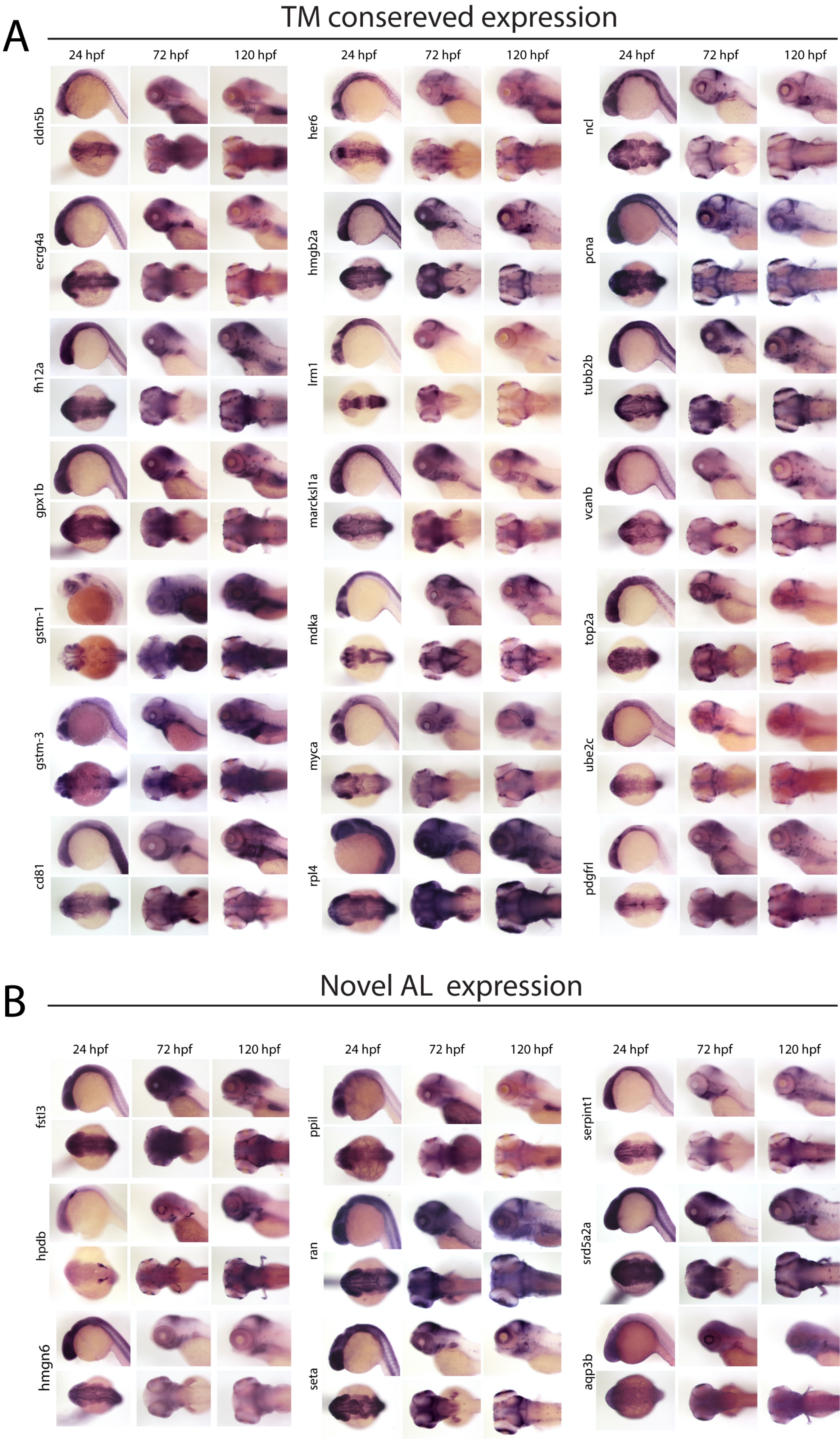

Supplement: Supplementary file 1 — Supplementary Information 1. [file 41598_2023_32212_MOESM1_ESM.pdf]
