# Supplementary material for: A temporal single cell transcriptome atlas of zebrafish anterior segment development
Source: Sci Rep. 2023 Apr 6;13:5656. doi: 10.1038/s41598-023-32212-4 (PMC10079958; doi:10.1038/s41598-023-32212-4)
Supplement: Supplementary file 2 — Supplementary Table S2. [file 41598_2023_32212_MOESM2_ESM.pdf]

TABLE 1

Gene	Forward Primer	Reverse Primer
adopa1	ATGAAGGTGTTTCTGGTCGTG	TAATACGACTCACTATAGGGGTTAGTGCTCTATTTTATT
ambp	ATGCGTGTGTTTTTGCTGTT	TAATACGACTCACTATAGGGAACTCATCGTCTCCGTTCCT
anxa1c	TCCAGAAATTGTTCAATCAC	TAATACGACTCACTATAGGGTTCAGCAGGTCGATTTCCGA
aqp3a	AAGCGTTCTGGATAAGCTTG	TAATACGACTCACTATAGGGTCATTCCTTGCTGGCGACGT
aqp3b	ATGGGAAGACAGAAGGTAAT	TAATACGACTCACTATAGGGTTATGCTGCATCTTTATCAG
bco1	ATGCAGTACGACTATGGCAA	TAATACGACTCACTATAGGGAACACAAATCTTGTGAACTT
cavin2a	ATGGGTGAAGACTCCTCTCA	TAATACGACTCACTATAGGGATATTGGGAACCTCCACTGC
cd81a	ATGGGCGTGGGCGTGGAAGG	TAATACGACTCACTATAGGGTTAATACACAGGGCTGTTCC
cdh5	ATGATGAAACAGTGTGCCAG	TAATACGACTCACTATAGGGGATTACTTCGGTTCTCTTCA
cldn5b	ATGGCAAATATGATTTCTGC	TAATACGACTCACTATAGGGTCAGACGTAGTTTCGTTTAT
cndp1	ATGATCAGCATTAGTGTGTT	TAATACGACTCACTATAGGGATGTCTGGCACCTGGCGGAT
col1a1b	ATGTTCAGCTTTGTGGATAT	TAATACGACTCACTATAGGGCAGGGTTACCAGGCTCACCA
col1a2	ATGCTCAGCTTTGTGGATAC	TAATACGACTCACTATAGGGCTCTCCAGTTGACCCTCTCT
col4a5	ATGAACTTAAAAAGTCTCGG	TAATACGACTCACTATAGGGTTGGTCCTATTCCACCTACA
col5a3b	ATGAAGATGAAGAGTTACCG	TAATACGACTCACTATAGGGATCTCCTATTCGTCCTGGGG
cthrc1a	ATGATGGGTACTAAACTGAC	TAATACGACTCACTATAGGGTCATTTTGGAAGCTCTTCAA
ctnnb2	ATGGCTAGCCAGGCTGACCT	TAATACGACTCACTATAGGGTCCACAGCAGCTTCTCATAG
cxcr4b	ATGGAATTTTACGATAGCAT	TAATACGACTCACTATAGGGCTAACTCGTCAGTGCACTGG
dcn	TCGTCAGCAACATGAAATCG	TAATACGACTCACTATAGGGCAATAGGGCACTGGATTACT
dct	ATGATCGCCACACAGCACTGG	TAATACGACTCACTATAGGGTTATGCTTCCCCTGAATATTT
defbl1	CCATCATCTGAAGAATCCAAC	TAATACGACTCACTATAGGGGTTTCTTAAATCAGTGGTGTT
ecrg4a	ATGCTTTCTGAAAAGTTTCA	TAATACGACTCACTATAGGGCTAGTAGTAGTCATAGTTGA
epcam	ATGAAGGTTTTAGTTGCCTT	TAATACGACTCACTATAGGGTTAAGAAATTGTCTCCATCT
fabp11a	ATCAAATCTCAATTTACAGC	TAATACGACTCACTATAGGGTAGAGAAGTAACATTTCACT
fhl2a	ATGACGGAGCGCTATGACTG	TAATACGACTCACTATAGGGTTAGATGTCTTTGCCACACT
fmoda	AAAGCCACTCCATATGCTACAGG	TAATACGACTCACTATAGCACACTGCAGAAACTGCCCAGC
frzb	ATGCAAAACATGTTTTCCTA	TAATACGACTCACTATAGGGTTTTCCAAGGACAGTTTAGTG
fstl3	ATGTGCTGGTTGCAGCAGGG	TAATACGACTCACTATAGGGTCTGCAGTGTCCATAGTGGC
galnt7	ATGAGGTTAAAGGTTGGATT	TAATACGACTCACTATAGGGTTTCCACAGGAGGCTCCAGT
gch2	AAGGCAGCAGAACTGAACAG	TAATACGACTCACTATAGGGTTTGGCCAGGGCCAGGAACT
gpx1b	ATGAACGAGCTGCACGAGCG	TAATACGACTCACTATAGGGTTATTTTGGGATGCTGAGGA
gstm.1	ATGGCAATGAAATTGGCTTA	TAATACGACTCACTATAGGGCCCATTTGGCCATCTTGTTG
gstm.3	ATGGCAATGAAGCTGGCATA	TAATACGACTCACTATAGGGCATTTGGCCATCTTGTTGTT
her6	ATGCCTGCCGATATCATGGA	TAATACGACTCACTATAGGGCTACCAAGGCCGCCAAACGG
hgd	GTGGATTTCGTATCGGGTCTA	TAATACGACTCACTATAGGGCTACTTGCTGCTGGGTTTCCA
hmgb2a	ATGGGTAAAGATCCAAATAA	TAATACGACTCACTATAGGGTTATTCGTCATCATCATCCT
hmgb2b	ATGGTGAAAGGAGACGTGAA	TAATACGACTCACTATAGGGCTACTCGTCATCTTCATCCT
hmgn2	CTGCAGCCAAGATGCCCAAAA	TAATACGACTCACTATAGGGTTTTATTCAAAACCATTTAAA
hmgn6	GTTCAGGACCCAACATGCCTA	TAATACGACTCACTATAGGGTGAGGAAAATTCCACCGTTTT
hpdb	ATGACATCGTACATTGACAA	TAATACGACTCACTATAGGGAACACAGTAGGTCTGTCCTG
hyal6	ATGGCGCTGATTGGATTTCT	TAATACGACTCACTATAGGGGACATTGATCACATAGTGAC
icn2	TACAGCCAGAAACATGAGTG	TAATACGACTCACTATAGGGTTTCAGTACAATAATATTAC
krt4	ATGTCAACCAGGTCTATCAC	TAATACGACTCACTATAGGGGCAATCTGAGCCTCCAAGTT
krt5	ATGTCTACTTCCTTCAAAAC	TAATACGACTCACTATAGGGGACTGCATCTCCTCAAACT
krt91	ATGTCTTCATACAGCCGTAG	TAATACGACTCACTATAGGGTTGGTAACCATTCAACATGT
lgals1l1	CTACCTGACACTTCATTTCA	TAATACGACTCACTATAGGGAGACTCAGTTTATTTGTGAA
lrrn1	ATGGCTAGAGGGACTTTCTT	TAATACGACTCACTATAGGGGAATCAGCCGTTGCCTGGTA
lum	ATGTTTGCTCTGGGATCCATTC	TAATACGACTCACTATAGGGCTATTCAAAGATGATCTCTGAG
lxn	ATGAAGACCGTCTGCTGGAC	TAATACGACTCACTATAGGGTATTGGTCTGGAGTGCTGGA
marcksl1a	ATGGGTGCTCAGTTGACTAA	TAATACGACTCACTATAGGGTCACTCTGCAGCCGGCTCCG
matn4	CTCCACCTACAGCAACTGGG	TAATACGACTCACTATAGGGCTTCATCACAGCGACAGTTA

mcm7	ATGGCCCCGAAGGATTATAC	TAATACGACTCACTATAGGGCGCCTGCTCCACTCCGCCCA
mdka	ATGCGGGGCCTGTTTTCCAC	TAATACGACTCACTATAGGGTTAGTTCCCTTTCCCCTTGC
myca	ATGCCGGTGAGTGCGAGTTT	TAATACGACTCACTATAGGGCTCGTTATTTGCGACCTCAG
myoc	CAGGTTCAGAGCAGTGCTAA	TAATACGACTCACTATAGGGGTATATAGCCCATAAGCCAT
ncl	ATGGTAAAGCTCGCTAAGGC	TAATACGACTCACTATAGGGTTGTCCAGTTTGACTGGCTG
nr2f1b	ATGGCCATGGTGGTGAGCGC	TAATACGACTCACTATAGGGGCAGCATATCCCTGATGAGC
nusap1	ATGGATTTAGACTCGCTAAA	TAATACGACTCACTATAGGGTGATGGTGCTAGCACTTCTG
pcna	ATGTTTGAGGCACGTCTGGT	TAATACGACTCACTATAGGGTTAGGAGGACTCTTCATCGA
pdgfrl	ATGAAGTTCTGGCTTTTCTT	TAATACGACTCACTATAGGGCTTATTCTTGGCAGTGCAGA
pfn1	ATGAGCTGGGACAGCTACAT	TAATACGACTCACTATAGGGTCACATGTTCATCTTCCTGA
pgm2	ATGGAGAATGGCCAGGGGAG	TAATACGACTCACTATAGGGAAGCACCTAATTCATTCCCA
phgdh	ATGGCTCCTATATCAGTCAA	TAATACGACTCACTATAGGGGATGTAACATGGAGCTGACT
pmela	TGCTACAGATGCATTAGCTAC	TAATACGACTCACTATAGGGTTAAACCGCTTGTATGTAAAA
pmelb	ATGAAGCTATTCTCTACAAT	TAATACGACTCACTATAGGGTGGAACAGTTTTCATTGCAG
pmp22a	ATGCTGGTCATTCTGTTTGCA	TAATACGACTCACTATAGGGTTCCCTTTGAGAATACACTTT
pnp4a	ATGCATAGTAAAGACCAAAT	TAATACGACTCACTATAGGGTCATGCCGTGTTGTTGTTGA
pnp5a	ATGTTTCCCGAGAGCAACAC	TAATACGACTCACTATAGGGTTAGGCGTAGTTGTTGTTGT
ppil1	ATGGCTGGAATACCGCCGGA	TAATACGACTCACTATAGGGTTAGCTGGGCAGATTCACTC
ran	ATGGCGGAGAACGAGCCACA	TAATACGACTCACTATAGGGTTAAAGATCGTCATCTTCAT
rpl9	ATGAAGACCATTCTCAGTAA	TAATACGACTCACTATAGGGTTAGTCCTCTTGCTGTTCCA
rrm2	ATGTCGTCCACTCGCTCTCC	TAATACGACTCACTATAGGGGAAATGTTCTCCATGAAGTC
scel	ATGTCTTTCACCGGAAAATC	TAATACGACTCACTATAGGGTTACAGTAACCACTTCAGTC
scinla	AGCACGGCATGGTGGATGATG	TAATACGACTCACTATAGGGTTAGTAACGACCACGGATGCG
serpinf1	ATGAAGAAGATAGTTCTGCT	TAATACGACTCACTATAGGGTGGCATACTCTGCGCCCTCT
seta	ATGTCTGCCTCAGCGGCTAA	TAATACGACTCACTATAGGGTCAGTCGTCATCACCTTCAT
si:ch211-133l5.7	CAGTGTGACTGCACTGTAAA	TAATACGACTCACTATAGGGTTAGAAATTGGTCTGCTTGG
si:ch211-243a20.3	ATGGCTGTTTCCACAACAAC	TAATACGACTCACTATAGGGTTCTGGATACACATAGGCTA
si:ch211-251b21.1	AACAGGCCTTAGTTTCATCAT	TAATACGACTCACTATAGGGAGAGGATTCTGGTGTTGCCAC
si:dkey-251i10.2	TATTGCAAATGAGCTATTGA	TAATACGACTCACTATAGGGAGCTTGACACCTGTGCTCTA
slc7a8a	ATGACGGATGGACCGAGACA	TAATACGACTCACTATAGGGGACTAGGCAGATGGCCTTCA
soul2	ATGGCGATGTATGTGGCTTC	TAATACGACTCACTATAGGGTCAGGGTGCATGGACCCAAA
sparc	ATGAGGGTTTGGATCTTCTTC	TAATACGACTCACTATAGGGGTGATATAGCTACAGTGTGAAC
srd5a2a	ATGCTCTGTCAGGAAAACAC	TAATACGACTCACTATAGGGTTATAGCAGGAAAGGGATCA
stmn1a	ATGGCTGCTACAAGTGACAT	TAATACGACTCACTATAGGGTCAGATTTCTTCACCATTGT
top2a	ATGATGACCATTGTGGGTCT	TAATACGACTCACTATAGGGATCAGGGCCAGACGTGTCCT
tspan36	ATGGATTGCGGAATTATAAC	TAATACGACTCACTATAGGGTCATGCGTACAGAGGTTGAT
tubb2b	GAAGTGTCTACTCCCAGCCA	TAATACGACTCACTATAGGGACATTGAGCATCTGCTCGTC
tyrp1b	ATGTGGAAGAGTGTGTGTTT	TAATACGACTCACTATAGGGTTACCCTGGGGAGCGCTGTA
ube2c	AAACTCTTGAAGAAATCGGT	TAATACGACTCACTATAGGGTTTAATTTCTGGGTGATCAA
vcanb	ATGATGTTGTTGGACGTGAA	TAATACGACTCACTATAGGGTAGGTGTTGTGGGTCCAGCT
vim	ATGGCCAGTCGAACAAGCAC	TAATACGACTCACTATAGGGTCATGTTGCGAATGTCGTCC
zgc:111983	ATGGAGTGGAGGATTATACT	TAATACGACTCACTATAGGGAAATGGAGCCATTCCTGAAC
zgc:158463	CCAGGAGTGGAGCCTGCGGC	TAATACGACTCACTATAGGGCCGCGCCAGGTTCACCGTTT
zgc:175088	ATGCCTGGAAACCTTATAAA	TAATACGACTCACTATAGGGTTAGACGTCTACGGTAACAG
zgc:92380	ATGTCATTCAGAAGCCCCTC	TAATACGACTCACTATAGGGTGAGCAGGGCCTCATAATCA
znfl1g	TCACCATGGCTGTTTCCACAA	TAATACGACTCACTATAGGGGTTCTGGATACACATAGGCTA
